# Modulatory effects of gut microbiome in cancer immunotherapy: A novel paradigm for blockade of immune checkpoint inhibitors

**DOI:** 10.1002/cam4.3694

**Published:** 2020-12-25

**Authors:** Sama Rezasoltani, Abbas Yadegar, Hamid Asadzadeh Aghdaei, Mohammad Reza Zali

**Affiliations:** ^1^ Foodborne and Waterborne Diseases Research Center Research Institute for Gastroenterology and Liver Diseases Shahid Beheshti University of Medical Sciences Tehran Iran; ^2^ Basic and Molecular Epidemiology of Gastrointestinal Disorders Research Center Research Institute for Gastroenterology and Liver Diseases Shahid Beheshti University of Medical Sciences Tehran Iran; ^3^ Gastroenterology and Liver Diseases Research Center Research Institute for Gastroenterology and Liver Diseases Shahid Beheshti University of Medical Sciences Tehran Iran

**Keywords:** cancer immunotherapy, gut microbiomarkers, gut microbiota, immune checkpoint inhibitors, oncomicrobes, PD‐1/PD‐L1 inhibitors

## Abstract

The human gastrointestinal (GI) tract harbors gut microbiome, which plays a crucial role in preserving homeostasis at the intestinal host‐microbial interface. Conversely, specific gut microbiota may be altered during various pathological conditions and produce a number of toxic compounds and oncoproteins, in turn, to induce both inflammatory response and carcinogenesis. Recently, promising findings have been documented toward the implementation of certain intestinal microbiome in the next era of cancer biology and cancer immunotherapy. Notably, intestinal microbiota can cooperate with immune checkpoint inhibitors (ICIs) of its host, especially in enhancing the efficacy of programmed death 1 (PD‐1) protein and its ligand programmed death ligand 1 (PD‐L1) blockade therapy for cancer. Herein, we review the dual function of gut microbiota in triggering GI cancers, its association with host immunity and its beneficial functions in modulation of cancer immunotherapy responses. Furthermore, we consider the significance of gut microbiota as a potential biomarker for predicting the efficacy of cancer immunotherapy. Finally, we summarize the relevant limitations that affect the effectiveness and clinical applications of gut microbiome in response to immunotherapy.

## Graphical abstract

In this review, we discuss the dual role of gut microbiome in triggering GI cancers, its association with host immunity and its beneficial functions in modulation of cancer immunotherapy responses. Furthermore, we consider the significance of gut microbiota as a potential biomarker for predicting the response to immunotherapy.

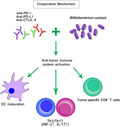

## 1 INTRODUCTION

The human gut microbiome is composed of a complex community of microbes, approximately 10^13^–10^14^ cells, which plays critical task in disease and health status.^1^ The intestinal microbiota consists of different microorganism types including archaea, bacteria, viruses, fungi, and protozoa that live on and inside various humans’ organs.^2^, ^3^ Different physiological acts can be attributed to gut microbiome, particularly inflammation, metabolism and immunity.^4^, ^5^

The immune system exploits different effector responses, cells and factors to eliminate pathogenic microbes and cancerous cells.^6^ Notably, gut microbiota destruction, identified as “dysbiosis,” has been correlated with a number of inflammatory conditions.^4^

Intestinal dysbiosis of healthy gut microbiota results in deterioration of mutualistic relationship and may associate with many diseases like metabolic syndrome, type 1 and type 2 diabetes, obesity, inflammatory bowel disease (IBD), irritable bowel syndrome (IBS), different types of cancers particularly gastrointestinal (GI) cancers (Table 1).^7^, ^8^

**TABLE 1:** Potential gut microbiomarkers associated with different types of GI cancers

Implicated microbiota	Type of cancer	Mode of action	Ref.
*Helicobacter pylori* ↑	Gastric cancer	Causing gastric infection Developing premalignant lesions Gastric atrophy, intestinal metaplasia, and dysplasia	16
*Fusobacterium nucleatum*↑	Colon cancer	Expanding myeloid derived immune cells in tumor microenvironment Mediating inflammation Resist in hypoxic tumor microenvironment and replicate Consumption of peptides in tumor environment to produce amino acid metabolites such as phenylalanine, methionyl, and formyl Activating Wnt/β‐catenin pathway and cell proliferation	7, 17, 18
*Streptococcus gallolyticus* ↑	Colon cancer	Enhancing inflammation and cell growth Contributing to overexpression of cyclooxygenase‐2 (PTGS2) during cancer Preventing apoptosis and promotion angiogenesis	7, 17
*Enterococcus faecalis* ↑	Colon cancer	Production of reactive oxygen species (ROS) to cause DNA damage Induction of chromosomal instability Producing extracellular superoxide anions as risk factors for colorectal carcinogenesis	7, 17
*Enterotoxogenic Bacteroides fragilis* (ETBF) ↑	Colon cancer	Cleavage of tumor suppressor protein Enhancing nuclear Wnt/β‐catenin signaling Enhancing cell growth and expression of c‐Myc proto‐oncogene Induction of NF‐kβ signaling and promoting secretion of cytokines from colon epithelial cell (CEC) Enhancing mucosal inflammation and CEC carcinogenesis	7, 17
Genotoxic *Escherichia coli* ↑	Colon cancer	Inducing double strand DNA breaks using the polyketide synthase (*pks*) island by colibactin	7, 17
*Porphyromonas gingivalis* ↑	Pancreatic cancer	Production of peptidylarginine deiminase (PAD) enzymes that can degrade arginine and result in K‐ras and p53 mutations	18
*Clostridium* spp.↑	Liver cancer	Inhibiting accumulation of hepatic natural killer T cells (NKT) Suppressing antitumor immunity against both primary and secondary liver tumors	19

Through changes in the intestinal lumen, certain commensal microbiota can quickly proliferate and acquire pathogenic features, such as vancomycin‐resistant *Enterococcus* or *Clostridium difficile*.^9^, ^10^ Gut microbiome complies all the prerequisites for representation as an endocrine body structure due to its plasticity and capability of producing various biologically functional components.^11^, ^12^ These metabolic by‐products and biologically active compounds like hormones that are released from this so‐called endocrine organ may circulate and disseminate to other body sites, and affect different pivotal biological procedures.^11^ Recent evidence strongly supports the important role of gut microbiota as a new therapeutic option in cancer treatment.^13^ Moreover, gut microbiota and their released metabolites have profound impacts on the development and response of peripheral immune system, and also it was demonstrated that can improve the therapeutic effectiveness of immune checkpoint inhibitors (ICIs) against cancerous cells.^14^, ^15^ Herein, we aimed to review the relationship between gut microbiota, host immune response and cancer immunotherapy, with a focus on the interaction of gut microbiota and ICIs. Also, we brought up the related pitfalls and challenges that may potentially affect the therapeutic capacity of microbiota in cancer immunotherapy. Furthermore, we discussed the possible role of chronic infections or inflammation that may interfere with cancer immunotherapy.

### 1.1 Gut microbiome and host immune system

Recent studies have suggested critical roles for the gut microbiome in the educating and development of major players of the host immunity through a complex microbiota‐immunity crosstalk in both homeostatic conditions and diseases.^20^, ^21^ These multifaceted dialogs not only authorize the immunological tolerance of commensal bacteria, but also enable the host immune cells to identify and begin an assault against microbial pathogens. Disturbance in the gut microbiome equilibrium is termed dysbiosis, which can result in considerable alterations in the taxonomical composition as well as the metagenomic functions of the gut microbiota and induce the overgrowth (blooming) of otherwise less abundant or potentially deleterious microbiota such as pathobionts.^22^, ^23^, ^24^ Once the dysbiosis occurred, it can directly or indirectly result in functional impairment of local, locoregional, and systemic immune responses leading to disintegration of epithelial barriers, and subsequently delivery of mucosa‐associated microbes and their components into the mesenteric lymph nodes (MLNs) and into the peripheral circulation.^23^ Moreover, dysbiosis‐associated inflammation can recruit neutrophils to the intestinal epithelium, alter the inflammatory cytokine and chemokine profiles, activate the T helper 17 (Th17) and effector T cells, which in turn may cause a negative feedback control of the gut microbiota.^25^, ^26^, ^27^

It has been well established that intestinal microbiota remarkably modulates and controls the development and operation of both the innate and adaptive immune systems. The microbial components and biomolecules, called microbe‐ or pathogen‐associated molecular patterns (MAMPs or PAMPs), and also their sensors named pattern recognition receptors (PRRs), are the key players which mediate the conversation between microbiota and host innate immune cells such as monocytes/macrophages, dendritic cells (DCs), and natural killer (NK) cells.^28^, ^29^, ^30^

In homeostatic conditions, the immune system orchestrates tolerance to beneficial intestinal microbiota such as *Bifidobacterium* and *Lactobacillus* species, while strongly reacts against the virulent microorganisms and opportunistic pathogens or pathobionts mainly through induction of the profound pro‐inflammatory responses.^31^, ^32^, ^33^, ^34^ Hence, there is a natural and prudent immunosurveillance system in the intestinal lumen which carefully monitors the microbial communities for maintaining the host‐microbiota mutualism and host defense. Moreover, normal intestinal flora can generate and synthesize various immunomodulatory compounds and metabolites such as short‐chain fatty acids (SCFAs) like propionate, acetate, and butyrate, and also secondary bile acids and ubiquitous bacterial fermentation products.^12^, ^35^ Of note, SCFAs act as effective inhibitors of histone deacetylases (HDACs) and lysine deacetylase (KDAC) in innate immune cells such as macrophages and DCs.^36^, ^37^, ^38^, ^39^ Furthermore, these bioactive agents are capable to interact with the over‐mentioned receptors on the immune cells and adjust their size, metabolic processes and functions which may result in host health benefits.^32^, ^40^ Thus, understanding the involved mechanisms behind the interactions between gut microbiome and immune system can be utilized to design and develop novel therapies to treat immune‐mediated and immune‐associated diseases.

### 1.2 Gut microbiota and NK cells

NK cells are key players of the innate immune system, and are characterized by the surface expression of marker CD56 and the lack of CD3 expression.^41^ This group of innate immune cells represents a heterogeneous subset of large granular lymphocytes, and constitutes nearly 5%–20% of all peripheral lymphocytes which are engaged in the clearance of virus‐infected cells and lysis of tumor cells.^42^, ^43^, ^44^ Beside their cytotoxic effector functions, NK cells are significantly involved in regulating the immune response by producing several cytokines and chemokines, mainly interferon‐γ (IFN‐γ) and tumor necrosis factor (TNF)‐α upon stimulation, to modulate other types of cells related to both the adaptive and innate immune responses.^44^, ^45^, ^46^ It has been shown that NK cells are not normally active killers but rather require to be completely activated in a process known as NK cell priming.^47^

Regarding the prominent function of NK cells in the biology of cancer, they obviously represented as forthcoming immunotherapeutic targets for the treatment of different malignancies, and a rising number of studies and ongoing clinical trials support the use of various therapeutic agents that target NK cell‐related pathways as cell‐based cancer immunotherapies.^48^, ^49^ The continuous existence of metabolites/products/ligands (e.g., LPS, peptidoglycan, SCFAs, and AhR ligands) originated from gut microbiota can induce the differentiation and activity of myeloid (monocytes/macrophages) lineage, including NK cells, and bone marrow progenitors, and also various groups of innate lymphoid cells (ILCs) through interacting with PRRs.^21^, ^50^, ^51^ Totally, NK cells play a critical role in response to gut microbial invasion, mainly via secretion of IFN‐γ, which can provoke recruitment of further NK cells from peripheral blood to augment the antimicrobial immune responses.^52^ These immune cells encounter a great number of antigens derived from commensal or potentially pathogenic microbes or pathobionts shaping the gut microbiome. Moreover, the crosstalk between NK cells and gut microbiota can lead to induction of adaptive T cell‐mediated immunity through interacting with professional antigen presenting cells (APCs) such as DCs.^52^, ^53^ It is also suggested that NK cells can evoke an intestinal inflammatory response during microbial invasions in the gut, which is irrespective of viral and tumor elimination. Also, these innate cells can exploit different toll‐like receptors (TLRs) to interact with various bacterial components like PAMPs, MAMPs, LPS, peptidoglycans, viral dsRNA, and DNA with CpG motifs to elicit inflammatory responses.^54^

NK cells have crucial roles in early defense against viral infections and a variety of tumors, and are involved in DC maturation, indicating a DC‐NK interplay which is of vital significance in antitumor immunity, and emphasizes the rationale for inspecting this crosstalk in the expansion of more efficacious cancer immunotherapies.^55^, ^56^, ^57^ On the contrary, certain strains of gut microbiota have been observed to modulate gut‐associated lymphoid tissue (GALT), enhancing the functional capability of innate immune response, activating DCs, and promoting NK cells though a direct cytochemical pathway by pathogens which invade the epithelial layer of the host gut.^42^, ^52^ It has been documented that NK cell priming and antiviral immune response were seriously compromised in germ‐free (GF) mice, which suggests that the presence of commensal microbiota is required to calibrate the function and priming of NK cells in GF mice.^47^ Furthermore, lactic acid bacteria (LAB) have been demonstrated to have considerable impact on maturation of DCs, therefore, activating NK cells.^58^ It has been shown that some strains of gut‐derived interleukin (IL)‐12‐inducing LAB can stimulate various subsets of DCs such as blood DCs and lymph node (LN) DCs, and activate NK cells to secrete IFN‐γ.^42^, ^59^, ^60^ Also, certain strains of probiotic bacteria that originated from a healthy gut microbiome, in particular *lactobacilli* and *bifidobacteria*, were reported to be involved in activation of NK cells, their functionality and cytotoxicity as a result of DC‐NK interplay.^42^, ^61^, ^62^ Taken together, these observations should represent a convincing rationale to explore the ligand‐receptor interactions between NK cells and healthy gut microbiota, which can be exploited as innovative targeted immunotherapies to help those with different conditions of intestinal inflammatory diseases associated with the gut immune system.

### 1.3 Role of oncomicrobes in cell proliferation and cancer initiation

**FIGURE 1:**
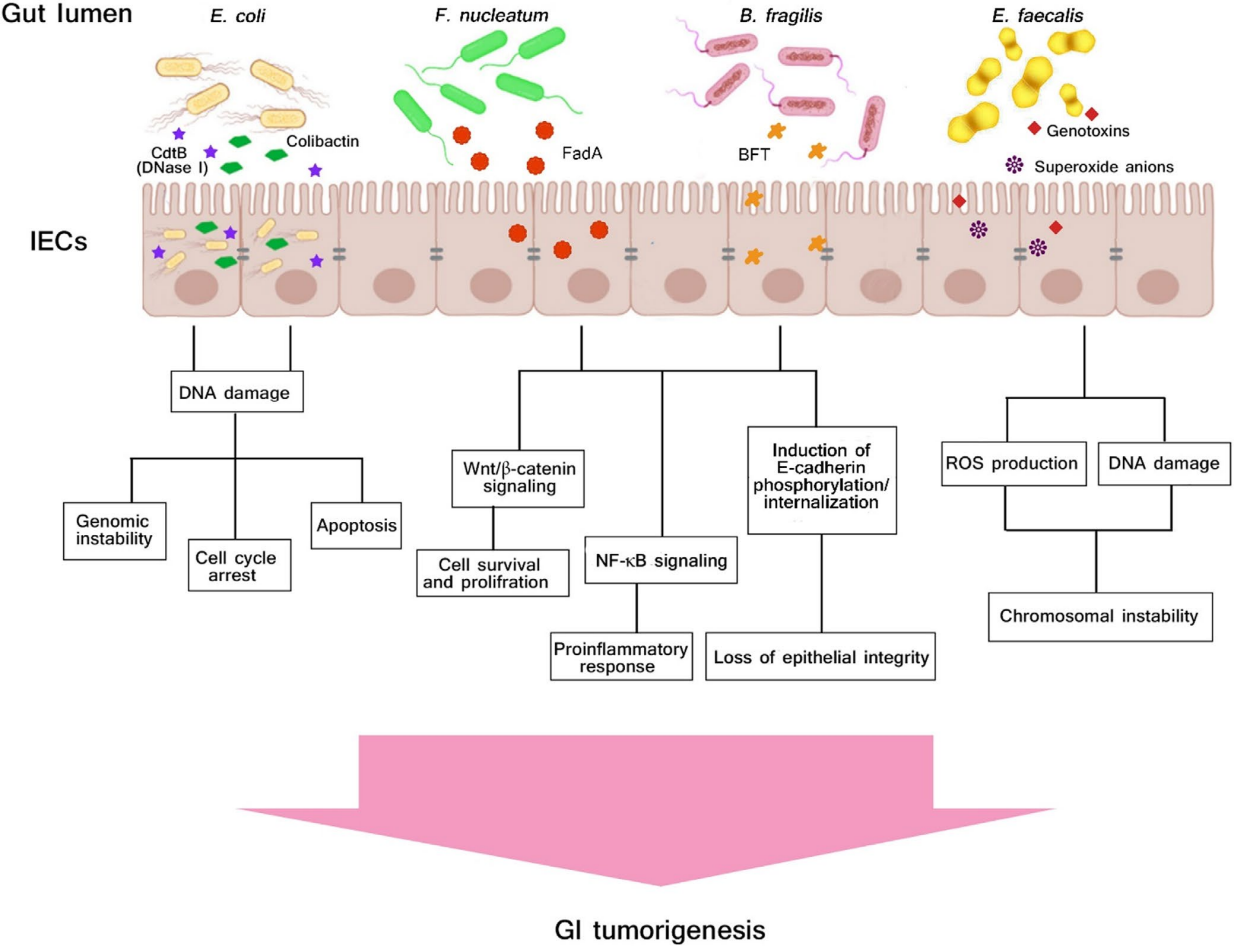
Schematic illustration of host‐microbiome interplay as potential trigger of gastrointestinal (GI) cancer. The mechanisms underlying the effects of certain gut microbiota and microbiome‐derived toxins and metabolites as potential triggers of GI tumorigenesis are described. Moreover, a series of pathways and process of carcinogenesis by which the gut microbiota may be involved in the genesis and development of GI tumorigenesis are depicted in the picture and mentioned in this review

Oncomicrobes contain microorganisms that induce direct DNA mutations and change host cellular signal transduction pathways. Until recently, oncomicrobes were mostly recognized to be viral agents such as human papillomavirus (HPV) that integrate their oncogenes inside the genetic content and frequently target the genes associated in various cancers.^63^ However, a few numbers of microbes are known as true oncomicrobes partially because of restrictions in recognizing microorganisms as irregular causes of cancers. The responsible microorganism may be depleted in the cancerous locations because it may have launched cellular injury via a “hit‐and‐run” strategy after a quick exposure to host cells.^63^, ^64^ In spite of lacking sufficient information associating cancer with specific bacterial species, various direct, and indirect plans are proposed by which they can induce different carcinogenesis pathways. Certain microbial species have evolved competitive approaches that contain the capacity to cause DNA damage of competing microorganisms. Also, such strategies can change host DNA material by forcing genetic alterations that may be involved in tumorigenesis. In addition, microbial DNA may be inserted into the host cellular genomes, especially the mitochondrial genetic content, via RNA intermediate molecules. These events occur mostly in cancerous tissues than normal adjacent tissues.^63^ Certain bacterial proteins are documented to induce signaling pathways involved in the host cellular cascades that modulate cell proliferation and stemness. For instance, Wnt/β‐catenin pathway, is aberrantly regulated via components generated by a number of bacteria, consisting of *Salmonella typhi*, *Fusobacterium nucleatum*, and *Helicobacter pylori*.^7^, ^63^, ^65^ DNA damage may also occur by bacterial toxins. For example, *Escherichia coli* producing colibactin, a newly identified substituted spirobicyclic molecule, induces crosslinking of double‐stranded DNA,^66^, ^67^ and cytolethal distending toxin (CDT) expressed by ƹ‐ and γ‐proteobacteria, demonstrates DNase activity and can directly induce DNA breaks^68^ (Figure 1).

## 2 CANCER IMMUNOTHERAPY

Cancer immunotherapy has recently attracted a great attention in the next era of cancer treatment. This new therapeutic strategy employs the host immune system to render antitumor effects against cancerous cells.^69^ Recently, ICIs are introduced as promising immunotherapeutic biomolecules, which have shown hopeful clinical outcomes in treatment of various cancers, as shown by monoclonal antibodies (mAbs) blocking cytotoxic T‐lymphocyte antigen‐4 (CTLA‐4), programmed cell death ligand 1 (PD‐L1) and programmed cell death protein 1 (PD‐1).^69^, ^70^ However, development of primary and acquired resistance throughout the duration of treatment period may decrease the ubiquitous clinical use of ICIs.^71^ Of note, selection of appropriate cases is critical to prevent subsequent resistance to such drugs and increase the efficacy of ICIs.^72^ Thus, robust attempts to combat the resistance to immunotherapy are extremely required.

It was observed that tumor cells expressing PD‐L1 induced apoptosis of co‐cultured activated effector T cells, and this process was inhibited by an antihuman PD‐L1 mAb.^73^, ^74^ In addition, the growth of murine tumors expressing PD‐L1 was blocked in syngeneic mice by the antimurine PD‐L1 mAb. Furthermore, similar findings were achieved through the examination of a variety cancer cells using animal models.^75^, ^76^, ^77^ These important findings opened the way to run several clinical trials exploiting mAbs targeting PD‐1, PD‐L1, and CTLA‐4 in cancer immunotherapy for different kinds of cancers. Presently, the U.S. Food and Drug Administration (FDA) have authorized the consumption of some mAbs including: cemiplimab (Libtayo), pembrolizumab (Keytruda), avelumab (Bavencio), atezolizumab (Tecentriq), durvalumab (Imfinzi), and nivolumab (Opdivo) for targeting PD‐1 and PD‐L1 in cancer immunotherapy.^78^, ^79^, ^80^ Also, ipilimumab (Yervoy) that targets the CTLA‐4 was demonstrated to function synergistically with nivolumab to induce T‐cell antitumor activity in melanoma and small lung cell carcinoma.^81^ Despite the obvious efficacy of PD‐L1, PD‐1, and CTLA‐4 suppression in cancer therapy, not all patients responded to these treatments. Therefore, practical strategies to enhance the effectiveness of cancer immunotherapy are demanded.^6^, ^82^

### 2.1 Interaction of PD‐1 and PD‐L1 in tumor microenvironment

In anticancer immunity, the immune system recognizes the tumor‐specific antigens expressed through gene mutations, and specific CD8^+^ cytotoxic T lymphocytes (CTLs) are recruited to the sites of tumor targeting the corresponding antigens.^83^ This certain cluster of effector CTLs identify the target tumor cells and induce programmed cell death of cancerous cells. Surprisingly, cancerous cells exploit different tactics to escape immune surveillance. For instance, they resist neutralizing effects of the antitumor CTLs by enhancing the expression level of PD‐L1 in tumor ecosystem.^75^, ^84^ Healthy host cells normally do not produce noticeable level of PD‐L1 on their surfaces, while PD‐L1 is significantly produced by tumor cells, immune, and nonimmune cells.^6^, ^85^ Interferon gamma (IFN‐γ) cytokine that is secreted by the infiltrating antitumor CTLs into tumor microenvironment, plays a key role in induction of PD‐L1 expression.^85^, ^86^ Moreover, some other cytokines like IL‐4, IL‐10, and TNF‐α can also upregulate the PD‐L1 expression.^87^, ^88^ The interplay between PD‐1 and PD‐L1 in tumor ecosystem capacitates the tumor cells to withstand the endogenous antitumor functions excreted from the host immune response.^87^ The interaction of PD‐L1 in tumor tissues with expressed PD‐1 on the activated T cells impairs the normal functions of effector T cells via multiple strategies, like induction of T cell programmed cell death, exhaustion, and anergy.^6^, ^69^, ^85^, ^86^ Recently, it was shown that crosstalk of PD‐1 with PD‐L1 expressed on tumor‐related macrophages prohibits the phagocytic capacity of macrophages against tumor cells.^89^ The significance of PD‐1 and PD‐L1 interplay in cancer cell escape promoted the utilization of such biomolecules as prominent therapeutic agents in immunotherapy of cancer.^87^, ^88^, ^89^

### 2.2 Gut microbiome and immunotherapy responses

Today, growing evidence has revealed that gut microbiome can play a key role in the modulation of immunotherapy responses in patients under treatment by immunotherapeutic drugs such as ICIs.^90^, ^91^, ^92^ The host response to ICIs, PD‐1/PD‐L1 blockade or CTLA‐4 inhibition, could be affected by the composition of intestinal microbiome.^93^, ^94^, ^95^ Upon PD‐1/PD‐L1 inhibition, mice with various intestinal microbial compositions have been shown to exert different responses to the anticancer immunotherapy.^93^ Gut microbiota analysis depicted that *bifidobacteria* were enhanced in mice with slow tumor growth, and exhibited promising responses to anti‐PD‐1 therapy. These favorable influences from mice having a more beneficial microbiome may be transported to other mice through fecal microbial transplantation (FMT).^92^ FMT is an effective strategy to normalize the intestinal microbiota which has already been employed in various clinical indications such as IBD, IBS, multiple sclerosis (MS), different type of cancers, and particularly in treatment of recurrent *Clostridioides* (formerly, *Clostridium*) *difficile* (rCDI) infection that do not response to conventional antimicrobial therapies.^96^, ^97^, ^98^, ^99^, ^100^, ^101^, ^102^, ^103^ FMT is defined as a therapeutic procedure that involves transplantation of the entire intestinal microbiota from a healthy donor into the intestinal tract of a patient to completely rebuild and normalize the structure and functionality of gut microbiome.^104^, ^105^, ^106^ In recent years, FMT also has attracted great interest to be applied along with cancer immunotherapy for solid tumor malignancies, specifically for improving the efficacy of ICIs.^92^ Together, due to enhancing the systemic and antitumor immune response in cancer patients, FMT could be administrated as a dramatic tool for the treatment of patients receiving ICIs.

Furthermore, the antitumor activity of PD‐L1 inhibition was increased when mice having an unpleasant gut microbiota were provided with oral probiotics containing *Bifidobacterium* bacteria.^107^ Such effects mostly arose from the maturation induction of dendritic cells DCs that lead to enhancement of cellular function of the tumor‐specific CD8^+^ T cells.^107^ Following CTLA‐4 blockade treatment, the richness of intestinal microbiota clearly differed in mice, as indicated by the relative enrichment of Burkholderiales and Bacteroidales and reduction of Clostridiales.^93^ Furthermore, mice oral feeding with *Bacteroides thetaiotaomicron*, *Bacteroides fragilis*, and *Burkholderia cepacia* increased the effectiveness of anti‐CTLA‐4 treatment by inducing T helper 1 (Th1) response and improving DC maturation. Nevertheless, consumption of broad‐spectrum antimicrobials in GF and specific‐pathogen‐free (SPF) mice significantly reduced the activity of anti‐CTLA‐4 treatment. This effect might be restored via FMT from individuals having predominant species of *Bacteroides*.^93^

Recent investigations have also confirmed the significance of intestinal microbiome in improving the effectiveness of cancer immunotherapy.^69^, ^92^ With PD‐1/PD‐L1 blockade therapies, the overall survival and the progression‐free survival (PFS) rates were significantly elevated in cases with epithelial tumors whom did not consume antimicrobials for routine purposes compared to cases with tumor that received antibiotics.^108^ This phenomenon declares that antibiotic usage may cause intestinal dysbiosis, hence, hindering the antitumor immunity and immune checkpoint blockade responses. Data obtained from the comprehensive metagenomic sequencing of fecal specimens from such cases demonstrated that responder participants to anti‐PD‐1 treatment had various compositions of intestinal microbiota, which were enriched in *Alistipes* and *Akkermansia*.^69^ Before PD‐1 blockade therapy, FMT was exploited in GF mice using fecal samples from responder donors that strengthened the immunity, while immune response of GF mice taking FMT from nonresponder donors was restored using *Akkermansia muciniphila* alone or in combination with *Enterococcus hirae*.^69^ Importantly, *A. muciniphila* was associated with enhanced infiltration of immune cells in tumor sites as CCR9^+^CXCR3^+^CD4^+^ T cells were migrated to the site of tumor, and CD4^+^ T cells to CD4^+^FoxP3^+^ T cells (Tregs) ratio was elevated.^91^, ^107^ In subjects with metastatic melanoma, the gut microbiome diversity was remarkably enhanced in responder cases to PD‐1 blockade treatment, and specific bacterial species were relatively more enriched, like *Faecalibacterium, Ruminococcaceae*, and Clostridiales.^63^, ^66^ However, nonresponder patients had less diverse population of gut microbiota and higher abundance of Bacteroidales.^92^ Analysis of the intestinal microbiome composition and the immunological patterns in the cancerous tissue showed that the expression of specific markers of cytotoxic T cells and antigen display were enhanced in individuals with beneficial intestinal microbiome in comparison with subjects having inappropriate gut microbiome.^92^ It was reported that tumor microenvironment of cases who responded to anti‐PD‐1 was abundant in *Collinsella aerofaciens*, *Bifidobacterium longum*, and *Enterococcus faecium*.^70^ Moreover, transfer of responder fecal specimens to GF mice positively reproduced the dominant phenotype, lower rate of tumor growth and promoted therapeutic impacts compared with mice that received nonresponder fecal samples. Consequently, these restorations of gut microbiota led to a rise in the overall population of CD8^+^ T cells and a reduction in Tregs in the tumor site.^95^

## 3 COMMENSAL MICROBIOTA AS POTENTIAL CONTROLLER OF CANCER IMMUNOTHERAPY

### 3.1 Beneficial microbiota

**FIGURE 2:**
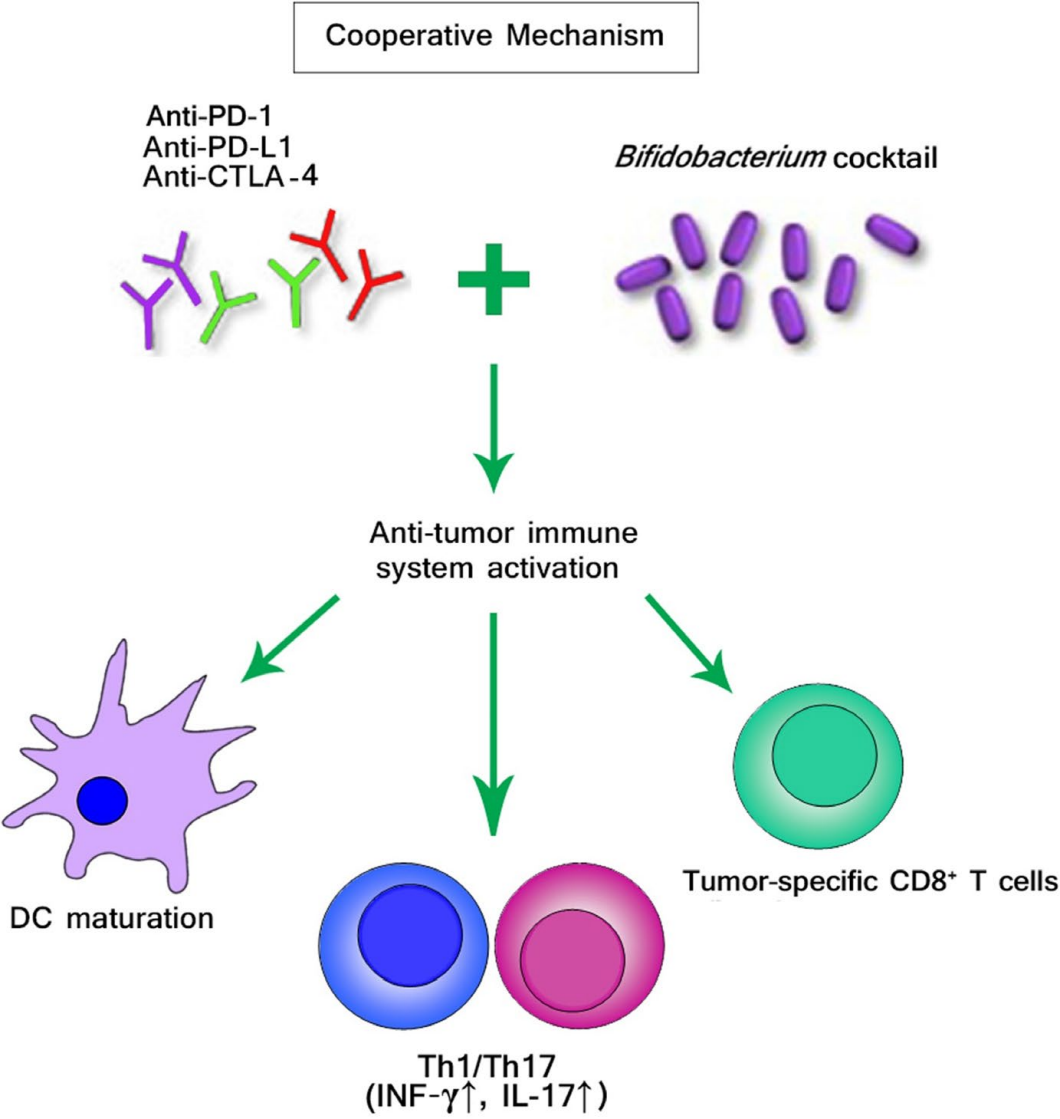
The putative effects of commensal microbiota on cancer immunotherapy. Certain beneficial microbial species are known to have a range of effects on host antitumor immune responses, and cancer immunotherapy. *Bifidobacterium* cocktail cooperates with immune checkpoint inhibitors (ICIs) blockade to promote and activate antitumor immunity. The identification of such cooperative mechanism may provide a novel and promising prospect for probiotic‐based therapies that could be integrated with cancer immunotherapy to ameliorate patient outcomes and even convert nonresponders

Results obtained from metagenomic studies using 16S ribosomal RNA (16S rRNA) sequencing revealed that *Bifidobacterium adolescentis*, *Bifidobacterium breve*, and *Bifidobacterium longum* were associated with increased efficacy of drugs used for cancer immunotherapy.^6^, ^107^ The function of these microbes in increasing defensive immune responses against tumors were subsequently evaluated by administering mice having solid tumors with *B. longum* and *B. breve* cocktail via oral feeding.^107^ In this experiment, *Bifidobacterium*‐treated mice demonstrated significant improvement in controlling tumor outgrowth as compared to untreated mice. It is hypothesized that *Bifidobacterium* cocktail can cooperate with immune checkpoint blockade to promote and activate antitumor immunity as depicted in Figure 2. Since *Bifidobacterium* species enhanced the anti‐melanoma effects by induction of innate immunity, the application of *Bifidobacterium* cocktail against tumor growth can be expanded to other types of cancers. Some of the typical bacterial species and viral agents that have been proposed to be positively or negatively linked to anti‐PD‐1 and anti‐PD‐L1 therapies are presented in Table 2.

**TABLE 2:** Microbial species and viral agents that are positively and negatively associated with PD‐1 and PD‐L1 blockade immunotherapy

Microbiota	Main effects on immunity	Potential effect on immunotherapy	Ref.
Beneficial microbiota	Enhanced the antitumor efficacy of PD‐L1 blockade, enhancement of DC maturation, improving activity of the tumor‐specific CD8^+^ T cells, increased IFN‐γ production	Effective	90, 107
*Bifidobacterium*			
*Bacteroides fragilis*, *Bacteroides thetaiotaomicron*, *Burkholderia cepacia*	Increased the efficacy of anti‐CTLA‐4 therapy by inducing Th1 response and promoting DC maturation, an increase in CD8^+^ T cells and a decrease in Tregs in the tumor environment	Effective	93
*Akkermansia muciniphila*	Enhanced the infiltration of immune cells in tumor site, as CCR9^+^CXCR3^+^CD4^+^ T cells were recruited to the tumor microenvironment and the ratio of CD4^+^ T cells to CD4^+^FoxP3^+^ T cells (Tregs) was enhanced	Effective	69
*Enterococcus hirae*	Enhanced IL‐12 secretion by DCs	Effective	110
Harmful microbiota	Increased host PD‐1 and PD‐L1 expression, higher level of pro‐inflammatory cytokines (TNF‐α), suppressed the proliferation of CD4+ T cells, the inhibitory effect can be blocked using antibodies PD‐L1	Ineffective	111
*Helicobacter pylori*			
HBV, HCV, HPV, EBV	Established chronic infections in humans and increased host PD‐1 or PD‐L1 expression	Ineffective	6

Abbreviations: CTLA‐4, cytotoxic T‐lymphocyte‐associated protein 4; DC, dendritic cell; EBV, Epstein‐Barr virus; HBV, hepatitis B virus; HCV, hepatitis C virus; HPV, human papillomavirus; IFN‐γ, interferon gamma; PD‐1, programmed death 1; PD‐L1, programmed death ligand 1; Th1, T helper type 1; TNF‐α, tumor necrosis factor‐α; Tregs, regulatory T cells.

Recently, another study examined the fecal specimens of metastatic melanoma cases prior to PD‐1 blockade cancer treatment, and demonstrated that abundance of *C. aerofaciens*, *E. faecium*, and *B. longum* were higher in the PD‐1 blockade immunotherapy responders, underpinning the antitumor actions of such microbes.^70^ Also, Frankel et al. proved that patients bearing melanoma who responded to ICIs were populated with *Bacteroides caccae*.^95^ Moreover, they presented that the kind of bacterial species which are increased within responders are most probably to be associated on the type of antibodies used against cancer immunotherapy. The gut microbiota of cases who responded to nivolumab (targeting PD‐1) were abundant with *Holdemania filiformis*, *Faecalibacterium prausnitzii* and *Bacteroides thetaiotaomicron*, while cases who responded to pembrolizumab (targeting PD‐1) were populated with *Dorea formicogenerans*. Nevertheless, the exact mechanisms behind these alterations are not well understood.^95^ Wargo et al. examined the human gut microbiome in participants with PD‐1 blockade therapy by whole genome shotgun sequencing and 16S rRNA metagenomics, and discovered that composition and diversity of bacteria in participants who responded to the immunotherapy were notably varied from that in participants who did not respond to the immunotherapy. The responders showed more diverse bacterial composition and higher number of Clostridiales, while the nonresponders were enriched with Bacteroidales.^109^ In another study, the effects of gut microbiome in anti‐PD‐1 therapy were investigated in patients bearing different cancers consisting of lung cancer, urothelial carcinoma, and renal cell carcinoma. They showed that cases who received antimicrobials prior or soon after beginning the anti‐PD‐1 treatment had diminished rate of survival, unless responders were enriched by *A*. *muciniphila*. They also observed that administration of *A. muciniphila* to SPF or GF mice was capable to rebuild the antitumor effects of anti‐PD‐1 therapy which was prevented by antibiotic usage.^69^ However, the precise mechanisms by which *A. muciniphila* enhances anti‐PD‐1 immunotherapy needs to be clarified.

### 3.2 Harmful microbiota

In recent studies, unmethylated CpG oligodeoxynucleotides that are frequently found in bacterial chromosomes, were documented to increase the antitumor function of CD8^+^ T cells by reducing PD‐1 expression through the IL‐12 cascade, proposing that intestinal microbiota that are positively related to anti‐PD‐1 and anti‐PD‐L1 immunotherapy may produce some metabolites which directly suppress PD‐1 and PD‐L1 expression.^111^, ^112^ Also, it seems likely that gut microbiota indirectly affect PD‐L1/PD‐1 expression via both systematically or locally mediating immune functions, thus, impacting the efficacy of anti‐PD‐1 and anti‐PD‐L1 treatment.^6^ For instance, polysaccharide A from *B. fragilis* was shown to stimulate Th1 cell responses.^113^ In addition, it was shown that oral feeding therapy with neomycin resulted in compromised immunity to infection by respiratory influenza virus, which was associated with significant reduction in the population of Gram‐positive bacteria in the intestine but not the nasal tract.^114^ Furthermore, there are well‐known microbial agents that are directly responsible for chronic infections in humans, some of them are identified to enhance PD‐1/PD‐L1 expression in host tissues.^115^, ^116^ For instance, *H. pylori infection* is one of the most prevalent human infections that can develop chronic active gastritis, peptic ulcers, and gastric adenocarcinoma.^117^, ^118^ Actually, *H. pylori*‐infected patients have a considerable secretion level of pro‐inflammatory cytokines, like TNF‐α ^119^, ^120^, ^121^ and higher production of PD‐L1 in gastric tissue as observed in a gastric cell line model of epithelial cells.^115^, ^119^, ^120^ Furthermore, *H. pylori* suppressed the proliferation of human CD4^+^ T cells originated from blood sample, however, such repressive impact can be inhibited by using antibodies against PD‐L1.^116^ Moreover, enhanced level of PD‐L1 expression was observed in gastric tissues of *H. pylori*‐infected patients, and also coculture of *H. pylori*‐infected primary gastric epithelial cells with T cells resulted in overexpression of PD‐L1 on gastric epithelial cells, which eventually led to induction of apoptosis in T cells. Taken together, these findings propose that *H. pylori* infection could induce a nonspecific suppression of circulating T cells, more importantly tumor‐specific T cells. Additionally, many viruses such as hepatitis B virus (HBV), hepatitis C virus (HCV), human papillomavirus (HPV), and Epstein‐Barr virus (EBV) are also capable to cause chronic infections and enhance human PD‐L1/PD‐1 expression.^121^, ^122^, ^123^, ^124^

### 3.3 Significance of gut microbiota as a promising biomarker to predict ICI efficacy

In the past few years, there has been rapidly rising interest in identifying potential biomarkers for predicting drug response to checkpoint blockade and providing prognostic data, basically in relation to cancer immunotherapy.^125^, ^126^ Along with the progress of the high‐throughput sequencing (HTS) technology, microarray tools and large‐scale analysis methods, a great number of biomarker identification strategies have been profoundly explored and have already resulted in promising outcomes.^127^, ^128^ Recent evidence conveys the potential application of intestinal microbiota as a predictive biomarker predicting the effectiveness of hematopoietic stem cell transplantation (HSCT), chemotherapy, and antitumor immunotherapy.^129^, ^130^, ^131^ It has also been shown that modulation of the intestinal microbiota may abolish inflammatory complications caused by ICI blockade therapy, thus, supporting the importance of microbial biomarkers and signatures in predicting the inflammatory adverse events (IAE) caused by cancer immunotherapy.^132^

Currently, the number of gut microbiome signatures as potential biomarkers that predict host response, and acquired resistance to ICI blockade treatment is rapidly expanding. In the recent years, substantial researches documented the synergistic cooperation of the certain gut microbiota with PD‐1/PD‐L1/CTLA‐4 inhibitors. For instance, *A. muciniphila*, *Alistipes indistinctus*, *Bacteroides*, *B. cepacia*, *D. formicigenerans*, *Parabacteroides merdae*/*distasonis*, *C. aerofaciens*, *Eubacterium* spp., *Veillonella parvula*, *Klebsiella pneumoniae*, *Bifidobacterium* spp., *Lactobacillus* spp., *Streptococcus parasanguinis*, *Blautia* spp., *E. hirae*, *E. faecium*, *H. filiformis*, *Faecalibacterium prausnitzii*, and *Gemmiger formicilis* as well as Ruminococcaceae family have been positively associated with response to checkpoint inhibition in the preclinical and clinical studies.^69^, ^70^, ^92^, ^94^, ^95^, ^130^ However, baseline enrichment in *B. thetaiotaomicron*, *Roseburia intestinalis*, *Anaerotruncus colihominis*, *Blautia obeum*, and some combination of antibiotics have been negatively correlated with response to anti‐PD‐1 and anti‐CTLA‐4 blockade and compromised the efficacy of immunotherapy.^69^, ^129^, ^133^ Furthermore, incorporation of gut microbiota‐derived proteomics, metabolomics, and genomics data paired with composition profiling of intestinal microbiota may lead to identification of unique metabolic signatures, which can be exploited as comprehensive biomarkers predicting the response to cancer immunotherapy. ^134^ However, there remain several critical issues such as inaccuracies in predicting the response to immunotherapy, that have to be conveyed in order to validate the application and efficacy of the intestinal microbiota as a prognostic and predictive biomarker for immunotherapy in the clinical practice.

### 3.4 Limitations and possible suggestions to enhance gut microbiota efficacy in cancer immunotherapy

In spite of promising exploitation of gut microbiota in the era of immunotherapy for cancer, there are as yet some issues and challenges which need to be considered. For example, the existence of unpleasant bacterial species in the intestinal tract can negatively influence the effectiveness of immunotherapy. Commonly, antibiotics are consumed to eliminate pathogenic bacterial species, but at the same time they may cause important risks owing to lack of specificity, particularly intestinal dysbiosis. On the contrary, use of probiotics in combination with prebiotics can synergistically help the intestinal colonization and augmentation of useful microbial species, and may have a booster effect to strengthen the host antitumor immune response. Moreover, the dietary fiber components may be metabolized and converted to biomolecules with immunomodulatory effects such as butyrate as a well‐known SCFA.^135^ Alternatively, bacteriophages (viruses that attack bacteria) have been mainly exploited in food industry to demolish pathogenic bacteria owing to their notable selectivity for certain bacterial agents.^136^

Recently, several studies have demonstrated that commensal gut microbiota can provide protection against the invasion of pathogenic microbes via colonization resistance mechanism, and also induction of the native or adaptive immune response though the immunomodulatory effects. This beneficial microbiota advocates colonization resistance through direct competing for nutrients and cellular attachment sites, and also produces various inhibitory metabolites which can restrict the overgrowth of the harmful microorganisms.^137^ Furthermore, despite the brilliant outcomes of FMT in the treatment of rCDI, administration of FMT could be a promising supplementary option beside immunotherapy against various types of human cancers.^96^, ^138^ However, application of FMT in cancer immunotherapy needs addressing several important issues, particularly the selection of an ideal donor, administration route, immune status of the recipient, and the types of cancer immunotherapeutic agents used. Moreover, it is noteworthy that still there are inconsistent findings between different studies regarding the impact of gut microbiota on cancer treatment.^69^, ^92^, ^93^, ^94^, ^102^ Hence, further studies including large cohorts, and clinical trials should be performed to assess the impact of gut microbiota on the effectiveness of ICIs.

## 4 CONCLUSIONS

The era of microbiota and cancer immunotherapy has recently been introduced and is still in its infancy. Currently, some primary reports of preclinical and clinical investigations on the function of gut microbiota in cancer immunotherapy have proposed it as an appropriate and alternative approach in war on cancer. It is worth noting to identify the specific microbiota and clarify their underlying mechanisms in the context of immune checkpoint blockade. Importantly, supplementation with specific probiotics or prebiotics and restoring the favorable intestinal microbiome by applying FMT or the prevention of the unfavorable bacteria by narrow‐spectrum antibiotics may improve the effectiveness of ICIs in tumor control. However, some problems and challenges stand to be addressed about how and when to manipulate intestinal microbiome to increase the potency of cancer immunotherapy. Finally, studying the potential variations in response to ICIs and exploring the possible hypotheses behind this therapeutic strategy should be accurately addressed by performing future studies in the setting of cancer immunotherapy, gut microbiome, metabolomics, proteomics, and genomics.

## CONFLICT OF INTEREST

The authors have no conflict of interest to disclose.

## AUTHOR CONTRIBUTIONS

SR and AY contributed to the literature review and wrote the draft of the manuscript. AY worked on concept and design of the study and interpreted the collected information. HAA and MRZ provided clinical advice and guidance for improving of the manuscript. AY critically revised the final version of the manuscript. All authors approved the final version of the manuscript and the authorship list.

## Funding information

This work was supported by Foodborne and Waterborne Diseases Research Center, Research Institute for Gastroenterology and Liver Diseases, Shahid Beheshti University of Medical Sciences, Tehran, Iran.

## Data Availability

All data generated or analyzed during this study are included in this published article.
